# Functional observation after morphine withdrawal: effects of SJP-005

**DOI:** 10.1007/s00213-021-05771-5

**Published:** 2021-02-08

**Authors:** Joris C. Verster, Andrew Scholey, Thomas A. Dahl, Jacqueline M. Iversen

**Affiliations:** 1 Division of Pharmacology, Utrecht Institute for Pharmaceutical Sciences (UIPS), Utrecht University, 3584CG, Utrecht, The Netherlands; 2 Centre for Human Psychopharmacology, Swinburne University, Melbourne, VIC 3122 Australia; 3 Psychopharmacology, Utrecht University, Po Box 80082, 3508TB Utrecht, Netherlands; 4 Sen-Jam Pharmaceutical, 223 Wall St., #130, Huntington, NY 11743 USA

**Keywords:** Opioid withdrawal, Opioid dependence, Opioid use disorder, Treatment, SJP-005, Lofexidine, Clonidine, Methadone, Buprenorphine

## Abstract

**Rationale and objective:**

SJP-005 (ketotifen and ibuprofen) is being developed as a potential new treatment for opioid withdrawal. Three studies were conducted to evaluate the early phase (acute, day 1) and late phase (days 2–12) effects of SJP-005 on discontinuation-induced morphine withdrawal.

**Methods:**

Sprague-Dawley rats received subcutaneous morphine twice daily for 18 days and ceased on day 19. Twice daily, oral dosages of placebo or SJP-005 (1 mg/kg ketotifen and 15 mg/kg ibuprofen) were administered starting 4 days before (study 1), 2 days before (study 2), or immediately after (study 3) morphine cessation. Functional observations were made up to 12 h after treatment cessation on day 19 (early phase), and immediately after treatment on days 20–30 (late phase). Treatment effects (mean overall score, and individual symptoms) were compared with placebo using ANOVA, and Tukey’s tests in case of multiple comparisons.

**Results:**

Across the studies, the number of withdrawal signs on day 19 (early phase) and days 20–30 (late phase) was lower with SJP-005 compared with placebo. The effects of SJP-005 when treatment was initiated 2 days before morphine cessation by discontinuation were most pronounced and statistically significant in the late phase (*F*_(1,18)_ = 14.10, *p* = 0.001). In particular, a significant reduction was observed in hypersensitivity to touch (*F*_(1,18)_ = 13.65, *p* = 0.002). A 50% reduction in withdrawal symptoms was observed 9.0 days after placebo versus 4.5 days after SJP-005. After 9.0 days, all withdrawal symptoms were absent in the SJP-005 group, while symptoms in the placebo group were still evident on day 18.

**Conclusion:**

Compared to placebo, SJP-005 significantly reduced the incidence and duration of discontinuation-induced morphine withdrawal symptoms when treatment was initiated 2 days before morphine cessation.

## Introduction

Opioids such as morphine, oxycodone and fentanyl are effective for reducing acute pain and as anesthesia during surgery. However, a serious side effect of opioids is their high potential for abuse and dependency, which can lead individuals to use them for prolonged periods of time increasing the risk of developing opioid use disorder. Pathways leading to opioid use disorder include the perception of inadequately controlled acute or chronic pain, the use of opioids to provide relief from emotional distress, and the recreational or non-medical opioid use (e.g., to obtain a high) (Stumbo et al. 2017). To ensure sufficient pain relief after sub-chronic use, prescribed medication dosages are often escalated (Hayes et al. 2020), treatments may be replaced by stronger opioids such as fentanyl, or individuals themselves may acquire additional (non-prescribed) opioids or alternatives such as heroin (Kolodny et al. 2015).

It is estimated that in the US about 3–5 million people suffer from opioid use disorder, representing a serious public health problem, referred to as the opioid epidemic (Schuckit 2016; NIDA 2019). In 2017, the opioid epidemic resulted in opioid use-related death of over 47,600 Americans (Scholl et al. 2018). These fatalities are accompanied by a devastating impact on human lives and the economy (The Council of Economic Advisers 2017). The opioid epidemic is an ongoing problem and is not limited to US. For example, increasing numbers of opioid use disorder are also reported in Europe (Verhamme and Bohnen 2019). Between 2008 and 2017, in The Netherlands, the number of opioid prescriptions nearly doubled from 4109 to 7489 per 100,000 inhabitants, and the number of opioid-related hospital admissions tripled from 2.5 to 7.8 per 100,000 inhabitants (Kalkman et al. 2019).

Individuals with opioid use disorder, who are physically dependent on opioids, experience intense withdrawal symptoms when opioid use is decreased or stopped. Opioid withdrawal symptoms are a constellation of subjective and clinical symptoms, including physiological effects that are often described as “flu-like.” Symptoms include feeling sick, gastrointestinal complaints, feeling of coldness, heart pounding, muscular tension, aches and pains, insomnia/trouble sleeping, sweating, restlessness, tremor, yawning, anxiety or irritability, gooseflesh skin, and runny nose or tearing. Because the intensity and duration of these symptoms is frequently severe, opioid drug use often persists beyond the patients’ interest in stopping, in part, to avoid withdrawal.

Current pharmacological treatment for opioid use disorder consists of methadone (a long-acting full opioid agonist), buprenorphine (a long-acting partial agonist), or naltrexone (an opioid antagonist). The aim of these treatments is to first replace current opioid use and/or abuse, and if preferred by an individual can be followed by gradual reduction until opioid use is ceased. These treatments are effective for this purpose. Patients are stabilized on a dosage regimen, after which the dosage can be reduced over time. However, although current pharmacological treatment reduces craving and withdrawal symptoms to some extent, opioid withdrawal symptoms are likely to occur each time the dosage is reduced. Moreover, to administer naltrexone an individual must first complete a 10-day period of opioid abstinence, referred to as the “induction hurdle.” This period of abstinence significantly increases the likelihood of experiencing opioid withdrawal symptoms, resulting in a high number of patients that fail to comply with treatment. Relapse to using opioid drugs is seen in 32–70% in patients within six month after treatment initiation and 72 to 88% of patients after 12–36 months (Strain et al. 2009; Chalana et al. 2015).

To prevent relapse by reducing the intensity and duration of opioid withdrawal symptoms, a variety of different medications are used, including alpha-2 adrenergic agonists (clonidine or lofexidine), and adjuvant agents that help mitigate individual symptoms of opioid withdrawal such as non-steroidal anti-inflammatory agents (NSAIDs) against pain (e.g., ibuprofen), antihistamines (e.g., hydroxyzine), anti-emetics against nausea (e.g., ondansetron), and anti-diarrheal drugs (e.g., loperamide). Double-blind, placebo-controlled randomized trials in humans have shown that both clonidine and lofexidine are effective in mitigating opioid withdrawal symptoms (Rehman et al. 2019; Alam et al. 2020). However, a high incidence of adverse effects is commonly reported for both treatments, including sedation, dry mouth, hypotension, drowsiness, and dizziness (Gowing et al. 2014; Hussain et al. 2015). A recent meta-analysis of studies that directly compared lofexidine and clonidine revealed that equivalent efficacy, with fewer adverse effects after lofexidine (Kuszmaul et al. 2020). Nevertheless, a recent study revealed no benefits of coadministering lofexidine or placebo to naltrexone in regard to compliance, opioid use, and overall opioid craving (Hermes et al. 2019). However, subjects who withdrawal from treatment due to medication intolerance was significantly higher when lofexidine was coadministered (Hermes et al. 2019).

Taken together, these findings suggest that there is a clear need for new safe and effective treatment options to aid opioid cessation. An ideal agent would not only reduce the constellation of acute withdrawal symptoms experienced shortly after opioid cessation but could also reduce behavioral symptoms such as opioid craving. Moreover, an agent that could reduce opioid craving, may also decrease relapse, which is itself associated with a high incidence of fatalities.

There is evidence that the central action of drugs of abuse is not restricted to neuronal effects but also involves glial cell activity producing several behavior changes that contribute to substance use disorder (Bachtell et al. 2017). Glial cells consist primarily of microglia and astrocytes. The action of morphine on neurons and glial cells results in activation of the Toll-like receptor 4 (TLR4) pathway, with the subsequent release of pro-inflammatory cytokines, and may be related to the undesirable side effects of morphine and related opioids, such as withdrawal, tolerance and dependence (Hutchinson et al. 2011).

The role of this pathway is supported by studies of TLR4 inhibitors. For example, ibudilast, a phosphodiesterase inhibitor with TLR4 inhibitor activity, decreases neurobiological markers indicative of the opioid-induced pro-inflammatory response, and attenuates both antagonist-precipitated and deprivation-induced morphine withdrawal in rodents (Hutchinson et al. 2009). In the first exploratory human trials, ibudilast decreased subjective ratings of opioid withdrawal symptoms (Cooper et al. 2016; Metz et al. 2017). The glial cell TLR4 pathway has also been found to be a contributor to drug reinforcement by increasing dopamine concentrations in the nucleus accumbens (Hutchinson et al. 2012). This suggests that inhibiting TLR4 may also reduce opioid craving. In addition, peroxisome proliferator-activated gamma receptor (PPAR-γ) agonists have been shown to reduce microglia pro-inflammatory cytokines. Recent studies show that stimulation of PPAR-γ, utilizing pioglitazone, a hypoglycemic agent, reduces heroin self-administration and reinstatement of heroin seeking (De Guglielmo et al. 2015, DE Guglielmo et al. 2017). A first clinical trial in humans found that pioglitazone did not reduce opioid withdrawal symptoms during buprenorphine taper (Schroeder et al. 2018). However, this study was ended prematurely due to slow enrollment, and did not have adequate statistical power to test study hypotheses. Therefore, more research is needed to examine the effects of PPAR-γ agonists in humans. Notwithstanding, all studies do suggest the involvement of a pro-inflammatory cytokine response possibly modulated by both the TLR4 pathway and by PPAR-γ activation, and these may serve as therapeutic targets to mitigate detrimental opioid withdrawal symptoms and behavior changes.

Here we describe the results of three rodent studies into the efficacy and safety of SJP-005, a new product that is being developed for the mitigation of opioid withdrawal symptoms to facilitate opioid discontinuation in opioid dependent adults. SJP-005 is a combination product that possesses TLR4 inhibition (the antihistamine ketotifen), with an NSAID that also possesses PPAR-γ agonist activity (ibuprofen). Because these two drugs have two different mechanisms of action, and both reduce pro-inflammatory cytokines, it was hypothesized that SJP-005 would significantly reduce opioid withdrawal symptoms.

## Methods

Three studies were conducted to investigate the effects of SJP-005 on morphine withdrawal by discontinuation (protocol numbers 0200RS128.002 (study 1), 0200RS128.001 (study 2), and 0200RS128.003 (study 3)). All studies were funded by Sen-Jam Pharmaceutical and conducted by Calvert Laboratories, Inc, Scott, PA (USA). The Calvert Institutional Animal Care and Use Committee (IACUC) approved the study protocols. Sprague-Dawley rats were obtained from Charles River Laboratories, Raleigh, NC (USA), and were experimentally naïve at the start of the studies. Animals were all adolescent males and approximately 7–8 weeks old at the time of first dosing.

### Housing

The treatment of Sprague-Dawley rats used in these studies was in accordance with Calvert SOPs, which adhere to the regulations outlined in the USDA Animal Welfare Act (9 CFR Parts 1, 2, and 3) and the conditions specified in the Guide for the Care and Use of Laboratory Animals (ILAR publication, NRC, 2011, The National Academies Press). Animals were group housed in compliance with the National Research Council “Guide for the Care and Use of Laboratory Animals.” For identification purposes, animals were ear-tagged and color coded. Assessments of pain and distress and the non-use of pain alleviating medication during the morphine withdrawal experiments were in accordance with “Criteria for Assessing Pain and Distress in Laboratory Animals.” Under controlled circumstances (12 h light/dark cycle, 20–26 °C and 30–70% humidity), animals were kept in a separate room specifically dedicated to conduct the studies, and had access to a Certified Rodent Diet (TEKLAD) or equivalent and water ad libitum. Animals were acclimated to the room for 8–9 days prior to first dosing. During this period, animals were monitored, and were replaced in case of observed abnormalities that suggest the possible development of infectious disease. Rats that were eligible to enter the study, i.e., those with a normal body weight and no clinical signs, were randomly assigned to one of the treatment groups. Treatment administration and functional observations were conducted in the same room.

### Study 1

Study 1 (0200RS128.002) was designed to evaluate the effects of SJP-0005 on discontinuation-induced morphine withdrawal, and its potential modification by ketotifen, ibuprofen, and SJP-005 (ketotifen and ibuprofen), when administered orally 4 days before and for 12 days after discontinuation-induced morphine withdrawal.

Four groups of *N* = 10 Sprague-Dawley rats received subcutaneous morphine twice daily (6 AM and 6 PM) for 18 days (starting at 3.2 mg/kg/injection, and increased by ¼ -log unit every 3 days to a final dose of 56.9 mg/kg/injection on days 16–18). Starting on day 15 until day 30, twice daily (6 AM and 6 PM), oral doses were administered of carboxymethyl cellulose placebo (group 1), ketotifen (1 mg/kg; group 2), ibuprofen (15 mg/kg; group 3), or SJP-005 (1 mg/kg ketotifen and 15 mg/kg ibuprofen; group 4). On day 19, morphine treatment was ceased. Functional observations were made on day 19, hourly from 1 to 12 h after dosing. On days 20–30, functional observations were made twice daily, immediately following dosing.

### Study 2

Study 2 (0200RS128.001) was designed to investigate the functional observation after morphine withdrawal by discontinuation or naltrexone administration, and its modification by SJP-005, when administered orally 2 days before and for 12 days after morphine withdrawal.

Four groups of *N* = 10 Sprague-Dawley rats received subcutaneous morphine twice daily (6 AM and 6 PM) for 18 days (starting at 3.2 mg/kg/injection, and increased by ¼ -log unit every 3 days to a final dose of 56.9 mg/kg/injection on days 16–18). Starting on day 17, group 1 and group 3 received a twice daily oral dose of carboxymethyl cellulose (placebo) and group 2 and group 4 a twice daily oral dose of SJP-005 (1 mg/kg ketotifen and 15 mg/kg ibuprofen), administered at 6 AM and 6 PM, until day 30. On day 19, morphine treatment was ceased in all groups. On day 19, group 1 and group 2 received an intraperitoneal injection of 0.32 mg/kg naltrexone, 2 h after the morning injection of morphine, whereas group 3 and group 4 had withdrawal by discontinuation.

In group 1 and group 2, on day 19, functional observations were made 15 and 30 min post-dose, then every 30 min post-dose until 3 h after dosing, and then hourly until 12 h after dosing. On days 20–30, functional observations were made twice daily, immediately following dosing. In group 3 and group 4, functional observations were made hourly on day 19, between 2 and 12 h following dosing. On days 20–30, functional observations were made twice daily, immediately following dosing.

### Study 3

Study 3 (0200RS128.003) was designed to evaluate the early phase (acute) and late phase (12 days) effects of SJP-005 on discontinuation-induced morphine withdrawal when SJP-005 is administered immediately following morphine discontinuation.

Two groups of *N* = 10 Sprague-Dawley rats received subcutaneous morphine twice daily (6 AM and 6 PM) for 18 days (starting at 3.2 mg/kg/injection, and increased by ¼ -log unit every 3 days to a final dose of 56.9 mg/kg/injection on days 16–18). On day 19, morphine treatment was ceased. Starting on day 19 until day 30, twice daily (6 AM and 6 PM) oral doses were administered of carboxymethyl cellulose placebo (group 1) or SJP-005 (1 mg/kg ketotifen and 15 mg/kg ibuprofen) (group 2). Morphine treatment was ceased on day 19. Two-hourly functional observations were made from 2 to 12 h following dosing on day 19 (early phase). On days 20–30 (late phase), functional observations were made twice daily, immediately following dosing.

### Treatment preparation and dosing

In all three studies, morphine was administered subcutaneously. Naltrexone was administered via intraperitoneal injection. Other treatments were administered directly to the oral gavage, without significant loss of solution from the oral cavity due to leaking or dripping. SJP-005 was administered as ketotifen and ibuprofen, one after the other.

### Functional observation

The duration of functional observation was 2 min. The rats were not video recorded, but were observed by the technicians at the designated times. Functional observation counts were recorded manually on paper. Within each study, all observations were made by the same investigator who was blind to the administered treatments. Observed opioid withdrawal signs included jumping, diarrhea, salivation, hypersensitivity to touch, rearing, wet dog shake, erection, lacrimation, vocalization, genital grooming, teeth grinding, ejaculation, and chromodacryorrhea. Most of the signs were spontaneous behaviors that were observed while the rats were in the Plexiglas® enclosures. There were a few observations where the animals were handled. For hypersensitivity to touch, the animals were picked up and evaluated for hypersensitivity to being handled. For each animal, during each assessment, it was recorded whether a withdrawal sign was observed or not, and a total score was computed for each group of animals. A total withdrawal symptom sum score was calculated for each group for day 19 (early phase), and days 20–30 (late phase), by adding together all observed events of the respective time period.

### Safety and adverse effects

Group food consumption and individual body weight were assessed daily, prior to the first dosing.

Adverse effects were monitored throughout the study. Animals displaying signs of pain or distress during the study were euthanized. Animals found dead or sacrificed for humane reasons prematurely during the study were subjected to necropsy and abnormalities were recorded. Dropouts were not replaced. After completion of the study, animals were euthanized by CO_2_ asphyxiation, and death was confirmed via thoracotomy.

### Statistical analysis

Analyses were conducted separately for day 19 (early phase) and days 20–30 (late phase). To be consistent across studies, for the analysis, for day 19 only hourly data collected between 2 and 12 h following dosing was used for the statistical analysis. For both periods, the total number of observed withdrawal symptoms was compared between treatments and placebo, using analysis of variance (ANOVA). Tukey’s tests were used for post hoc comparisons. Differences from placebo were considered statistically significant if *p* < 0.05.

Trendline data was analyzed using Statgraphics Centurion, version 19. For each treatment group, linear regression models were computed for the daily total number of withdrawal symptoms scores of the late phase, and trendlines were plotted. The equation of the trendlines (*y* = *ax* + *b*) and intercept and slope were determined. Using the corresponding equations, the daily withdrawal sum scores (*x*) were entered to determine the day at which zero symptoms are expected according to the trendline. Intercepts and slopes of the trendlines of different groups were compared using ANOVA. Differences were considered statistically significant if *p* < 0.05. Data on body weight and food intake were compared using ANOVA. Tukey’s tests were used for post hoc comparisons. Differences were considered statistically significant if *p* < 0.05.

## Results

### Study 1

In study 1, treatment administration started 4 days before discontinuation-induced morphine withdrawal.

Functional observations for the four groups are summarized in Table 1 (early phase, day 19) and Table 2 (late phase, days 20–30).

**Table 1:** Functional observations after withdrawal by discontinuation: early phase (T4)

Functional observation	Group 1 (placebo)	Group 2 (ketotifen)	Group 3 (ibuprofen)	Group 4 (SJP-005)
Jumping	0.0 (0.0)	0.0 (0.0)	0.0 (0.0)	0.0 (0.0)
Diarrhea	0.0 (0.0)	0.0 (0.0)	0.1 (0.3)	0.0 (0.0)
Salivation	0.0 (0.0)	0.0 (0.0)	0.0 (0.0)	0.0 (0.0)
Hypersensitivity to touch	4.9 (2.2)	4.3 (2.9)	3.7 (2.9)	4.6 (3.7)
Rearing	0.0 (0.0)	0.0 (0.0)	0.3 (0.7)	0.2 (0.6)
Wet dog shake	0.0 (0.0)	0.0 (0.0)	0.5 (1.1)	0.3 (0.9)
Erection	2.9 (2.4)	0.7 (0.8)	1.8 (1.50	2.2 (2.0)
Lacrimation	0.0 (0.0)	0.0 (0.0)	0.0 (0.0)	0.0 (0.0)
Vocalization	6.5 (2.4)	4.6 (2.8)	4.2 (2.7)	5.7 (3.5)
Genital grooming	0.0 (0.0)	0.0 (0.0)	0.0 (0.0)	0.0 (0.0)
Teeth grinding	0.0 (0.0)	0.0 (0.0)	0.0 (0.0)	0.0 (0.0)
Ejaculation	0.1 (0.3)	0.0 (0.0)	0.0 (0.0)	0.0 (0.0)
Chromodacryorrhea	0.0 (0.0)	0.0 (0.0)	0.0 (0.0)	0.0 (0.0)
Total symptom score	14.4 (6.0)	9.6 (5.8)	10.6 (7.2)	13.0 (9.4)

Treatment administration started 4 days before discontinuation-induced morphine withdrawal (T4). Mean (SD) of all assessments on day 19 are presented

**Table 2:** Functional observations after withdrawal by discontinuation: late phase (T4)

Functional observation	Group 1 (placebo)	Group 2 (ketotifen)	Group 3 (ibuprofen)	Group 4 (SJP-005)^1^
Jumping	0.2 (0.4)	0.3 (0.9)	0.1 (0.3)	0.0 (0.0)
Diarrhea	0.9 (1.0)	0.4 (0.8)	0.9 (1.0)	0.8 (1.0)
Salivation	0.0 (0.0)	0.0 (0.0)	0.0 (0.0)	0.0 (0.0)
Hypersensitivity to touch	9.3 (3.1)	12.3 (4.8)	9.3 (4.2)	8.3 (2.3)
Rearing	0.0 (0.0)	0.1 (0.3)	0.2 (0.4)	0.1 (0.4)
Wet dog shake	0.2 (0.4)	0.0 (0.0)	0.1 (0.3)	0.0 (0.0)
Erection	2.6 (1.6)	1.7 (1.3)	2.8 (2.1)	1.5 (0.8)
Lacrimation	0.0 (0.0)	0.0 (0.0)	0.0 (0.0)	0.0 (0.0)
Vocalization	12.3 (3.7)	15.5 (5.1)	14.2 (3.4)	10.3 (4.3)
Genital grooming	0.0 (0.0)	0.0 (0.0)	0.0 (0.0)	0.0 (0.0)
Teeth grinding	0.0 (0.0)	0.0 (0.0)	0.0 (0.0)	0.0 (0.0)
Ejaculation	0.6 (1.0)	0.0 (0.0)	0.3 (0.5)	0.9 (0.8)
Chromodacryorrhea	0.0 (0.0)	0.0 (0.0)	0.0 (0.0)	0.0 (0.0)
Total symptom score	26.3 (8.0)	30.3 (11.4)	27.9 (8.6)	21.8 (7.0)

Treatment administration started 4 days before discontinuation-induced morphine withdrawal (T4). Mean (SD) of assessments on days 20–30 are presented

^1^*N* = 8

In the placebo group, morphine withdrawal on day 19 was associated by clear increase of discontinuation symptoms such as vocalization, hypersensitivity to touch, and number of erections. ANOVA revealed no significant differences between the groups in the mean number of withdrawal symptoms (*F*_(3,36)_ = 0.92, *p* = 0.442). Also, withdrawal scores of individual symptoms did not differ significantly between the treatments.

During days 20–30, no significant differences between the treatments were found for the total symptom score (*F*_(3,36)_ = 1.40, *p* = 0.259). Withdrawal scores of individual symptoms did also not differ significantly between the treatments.

**Fig. 1:**
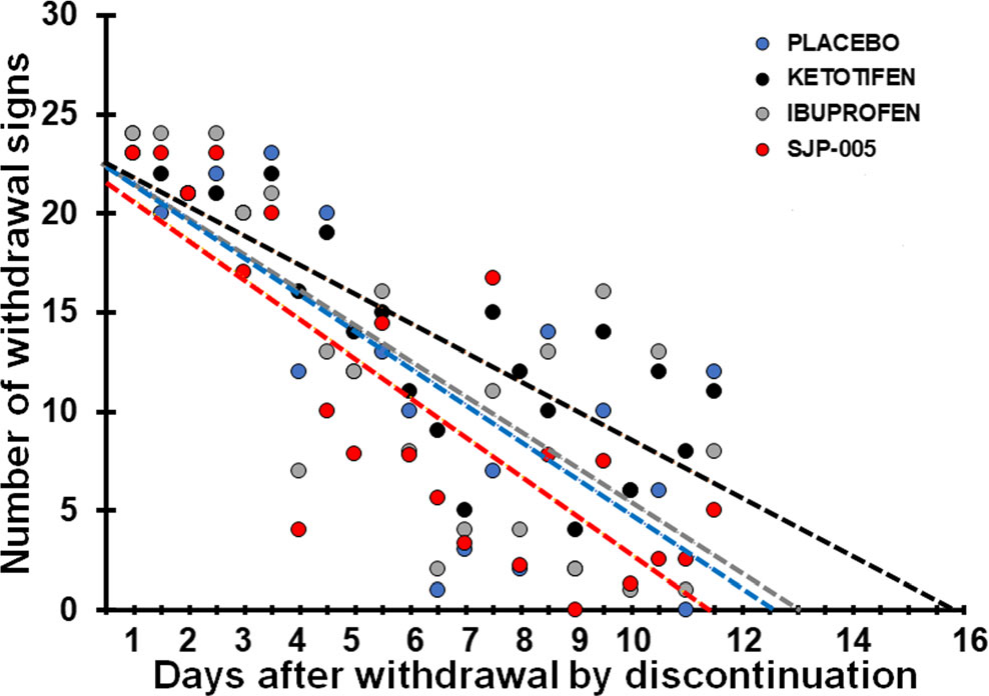
Number of withdrawal signs after morphine withdrawal by discontinuation (T4). Treatment administration started 4 days before morphine cessation (T4). Dots represent the total number of observed withdrawal signs for each group (*N* = 10) for each day; broken lines are lines of best fit for each condition

Regression analysis was conducted on the total number of withdrawal scores observed after morphine withdrawal by discontinuation during the late phase (days 20–30). Linear trend lines were computed for the placebo group (*y* = − 1.87*x* + 23.5), ketotifen group (*y* = − 1.47*x* + 23.3), ibuprofen group (*y* = − 1.77*x* + 23.1), and the SJP-005 group (*y* = − 1.99*x* + 22.7). According to the trendline equations, a 50% reduction in withdrawal symptoms was found 6.3 days after placebo, 7.9 days after ketotifen, 6.5 days after ibuprofen, and 5.7 days after SJP-005. After 11.4 days, all withdrawal symptoms were absent after SJP-005, while withdrawal symptoms are likely to be expected up to 12.6 days after placebo, 13.1 days after ibuprofen, and 15.9 days after ketotifen (see Fig. 1). ANOVA revealed no significant differences between the intercepts and slopes of the treatment groups and placebo group trendline.

### Study 2

In study 2, treatment administration (SJP-005 vs placebo) started 2 days before naltrexone-precipitated withdrawal (groups 1 and 2) or discontinuation-induced morphine withdrawal (groups 3 and 4). Functional observations for the two naltrexone-precipitated withdrawal groups are summarized in Table 3.

**Table 3:** Functional observations after naltrexone-precipitated withdrawal (T2)

	Day 19		Days 20–30	
Functional observation	Group 1 (placebo)	Group 2 (SJP-005)	Group 1 (placebo)	Group 2 (SJP-005)
Jumping	0.0 (0.0)	0.0 (0.0)	0.0 (0.0)	0.0 (0.0)
Diarrhea	2.2 (0.4)	1.8 (0.8)	0.0 (0.0)	0.0 (0.0)
Salivation	0.2 (0.4)	0.5 (0.5)	0.0 (0.0)	0.0 (0.0)
Hypersensitivity to touch	4.6 (2.2)	3.5 (1.2)	10.2 (4.6)	7.5 (2.0)
Rearing	0.0 (0.0)	0.0 (0.0)	0.0 (0.0)	0.0 (0.0)
Wet dog shake	1.6 (1.9)	1.6 (2.0)	0.1 (0.3)	0.2 (0.4)
Erection	0.7 (0.9)	2.1 (3.2)	0.0 (0.0)	0.2 (0.4)
Lacrimation	0.0 (0.0)	0.0 (0.0)	0.0 (0.0)	0.0 (0.0)
Vocalization	0.0 (0.0)	0.0 (0.0)	0.0 (0.0)	0.0 (0.0)
Genital grooming	2.4 (1.6)	1.4 (1.1)	0.2 (0.4)	0.4 (0.5)
Teeth grinding	1.0 (1.1)	0.3 (0.5)	0.0 (0.0)	0.0 (0.0)
Ejaculation	0.4 (1.0)	0.2 (0.4)	0.0 (0.0)	0.0 (0.0)
Chromodacryorrhea	0.2 (0.6)	0.5 (0.8)	0.0 (0.0)	0.0 (0.0)
Total symptom score	13.3 (5.5)	11.9 (4.4)	10.5 (4.3)	8.3 (2.3)

Treatment administration started 2 days before naltrexone-precipitated withdrawal (T2). Mean (SD) of all assessments are presented

On day 19, the incidence of naltrexone-precipitated withdrawal signs after placebo or SJP-005 did not significantly differ between the groups (*F*_(1,18)_ = 0.39, *p* = 0.540). No significant differences between the two groups were found for individual symptoms. During days 20–30, withdrawal signs abated over time after both placebo and SJP-005.The difference between the placebo and SJP-005 group was not statistically significant (*F*_(1,18)_ = 2.05, *p* = 0.169), and no significant differences between the two groups were found for individual symptoms.

**Fig. 2:**
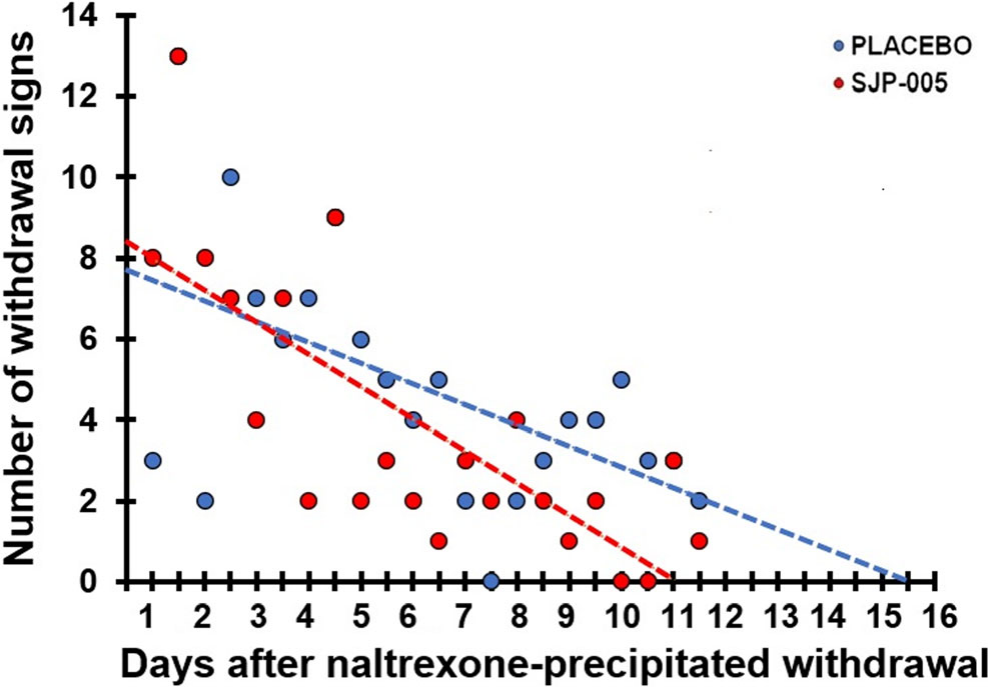
Number of withdrawal signs after naltrexone-precipitated withdrawal (T2). Treatment administration started 2 days before naltrexone-precipitated withdrawal (T2). Dots represent the total number of observed withdrawal signs for each group (*N* = 10) for each day; broken lines are lines of best fit for each condition

Regression analysis was conducted on the total number of withdrawal scores observed after naltrexone-precipitated withdrawal during the late phase (days 20–30). Linear trend lines were computed for the placebo group (*y* = − 0.51*x* + 8.0) and SJP-005 group (*y* = − 0.80*x* + 8.8). According to the trendline equations, after 11.0 days, all withdrawal symptoms are absent after SJP-005, while withdrawal symptoms are likely to be expected up to 15.7 days in the placebo group (see Fig. 2). ANOVA revealed that the intercepts (*F*_(1,2)_ = 1.70, *p* = 0.200) and slopes (*F*_(1,2)_ = 1.53, *p* = 0.224) of the trendlines did not significantly differ between the groups.

Functional observations for the two withdrawal by discontinuation groups are summarized in Table 4.

**Table 4:** Functional observations after withdrawal by discontinuation (T2)

	Day 19		Days 20–30	
Functional observation	Group 3 (placebo)	Group 4 (SJP-005)	Group 3 (placebo)	Group 4 (SJP-005)
Jumping	0.0 (0.0)	0.0 (0.0)	0.0 (0.0)	0.0 (0.0)
Diarrhea	0.4 (0.7)	0.0 (0.0)	0.1 (0.3)	0.1 (0.3)
Salivation	0.2 (0.4)	0.0 (0.0)	0.0 (0.0)	0.0 (0.0)
Hypersensitivity to touch	4.3 (2.2)	4.7 (3.0)	11.8 (5.1)	5.4 (1.9) *
Rearing	0.0 (0.0)	0.0 (0.0)	0.0 (0.0)	0.0 (0.0)
Wet dog shake	2.4 (2.2)	1.4 (1.6)	0.2 (0.4)	0.2 (0.4)
Erection	0.4 (0.7)	0.2 (0.6)	0.0 (0.0)	0.2 (0.6)
Lacrimation	0.0 (0.0)	0.0 (0.0)	0.0 (0.0)	0.0 (0.0)
Vocalization	0.0 (0.0)	0.1 (0.3)	0.0 (0.0)	0.0 (0.0)
Genital grooming	0.6 (0.8)	0.4 (0.7)	0.1 (0.3)	0.2 (0.4)
Teeth grinding	0.3 (0.5)	0.1 (0.3)	0.0 (0.0)	0.0 (0.0)
Ejaculation	0.0 (0.0)	0.1 (0.3)	0.0 (0.0)	0.0 (0.0)
Chromodacryorrhea	0.0 (0.0)	0.0 (0.0)	0.0 (0.0)	0.0 (0.0)
Total symptom score	8.7 (2.4)	7.0 (4.8)	12.4 (5.0)	6.1 (1.9) *

Treatment administration started 2 days before morphine withdrawal by discontinuation (T2). Mean (SD) of all assessments are presented. Significant differences (*p* < 0.05) between SJP-005 and placebo are indicated by *

After withdrawal by discontinuation, withdrawal signs on day 19 were lower after SJP-005 compared to placebo, but the reduction in observed withdrawal symptoms was not statistically significant (*F*_(1,18)_ = 1.02, *p* = 0.327). Also, scores of none of the individual symptoms differed significantly between the placebo and SJP-005 groups. During days 20–30, withdrawal signs significantly abated after both placebo and SJP-005. The difference between the placebo group and SJP-005 group was statistically significant (*F*_(1,18)_ = 14.10, *p* = 0.001). Compared to placebo, after SJP-005 a significant reduction was observed for hypersensitivity to touch (*F*_(1,18)_ = 13.65, *p* = 0.002). Other withdrawal signs were infrequently reported, and for these symptoms no significant differences were found between the placebo and SJP-005 groups.

**Fig. 3:**
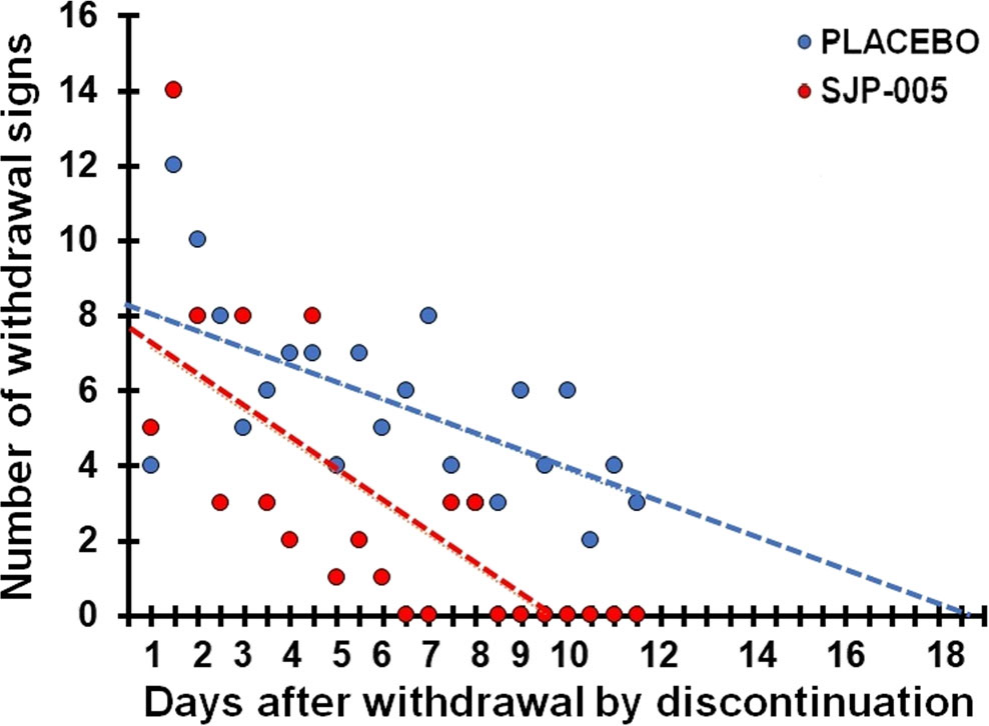
Number of withdrawal signs after morphine withdrawal by discontinuation (T2). Treatment administration started 2 days before morphine withdrawal by discontinuation (T2). Dots represent the total number of observed withdrawal signs for each group (*N* = 10) for each day; broken lines are lines of best fit for each condition.

Regression analysis was conducted on the total number of withdrawal scores observed after morphine withdrawal by discontinuation during the late phase (days 20–30). Linear trend lines were computed for the placebo group (*y* = − 0.46*x* + 8.3) and SJP-005 group (*y* = − 0.84*x* + 7.6). According to the trendline equations, a 50% reduction in withdrawal symptoms was found 9.0 days after placebo versus 4.5 days after SJP-005. After 9.0 days, all withdrawal symptoms are absent after SJP-005, while withdrawal symptoms are likely to be expected up to 18.0 days in the placebo group (see Fig. 3). ANOVA revealed that the intercepts of the trendlines differ significantly from each other (*F*_(1,2)_ = 16.95, *p* = 0.0002). The slopes did not significantly differ between the SJP005 group and placebo group (*F*_(1,2)_ = 2.84, *p* = 0.099).

### Study 3

In study 3, treatment administration (SJP-005 vs placebo) started on the same day (day 19) as discontinuation-induced morphine withdrawal. Functional observations for the two groups are summarized in Table 5.

**Table 5:** Functional observations after withdrawal by discontinuation (T0)

	Day 19		Days 20–30	
Functional observation	Group 1 (placebo)	Group 2 (SJP-005)	Group 1 (placebo)	Group 2 (SJP-005)
Jumping	0.0 (0.0)	0.0 (0.0)	0.0 (0.0)	0.0 (0.0)
Diarrhea	0.0 (0.0)	0.0 (0.0)	0.1 (0.3)	0.0 (0.0)
Salivation	0.0 (0.0)	0.0 (0.0)	0.0 (0.0)	0.0 (0.0)
Hypersensitivity to touch	0.9 (1.1)	0.9 (1.0)	10.7 (8.0)	8.8 (6.6)
Rearing	0.0 (0.0)	0.0 (0.0)	1.2 (1.1)	1.0 (1.3)
Wet dog shake	0.4 (0.5)	0.1 (0.3)	0.0 (0.0)	0.0 (0.0)
Erection	0.2 (0.4)	0.1 (0.3)	1.2 (1.3)	0.2 (0.4) *
Lacrimation	0.0 (0.0)	0.0 (0.0)	0.0 (0.0)	0.0 (0.0)
Vocalization	4.4 (3.5)	3.7 (3.3)	7.3 (5.3)	5.6 (4.9)
Genital grooming	0.0 (0.0)	0.0 (0.0)	0.0 (0.0)	0.0 (0.0)
Teeth grinding	0.0 (0.0)	0.0 (0.0)	0.0 (0.0)	0.0 (0.0)
Ejaculation	0.0 (0.0)	0.0 (0.0)	0.2 (0.4)	0.1 (0.3)
Chromodacryorrhea	0.0 (0.0)	0.0 (0.0)	0.0 (0.0)	0.0 (0.0)
Total symptom score	4.4. (3.5)	3.7 (3.3)	15.0 (4.7)	12.6 (4.0)

Treatment administration started on the same day (day 19) as discontinuation-induced morphine withdrawal (T0). Mean (SD) of all assessments are presented. Significant differences (*p* < 0.05) between SJP-005 and placebo are indicated by *

On day 19, no significant differences in total withdrawal sum scores was found between the SJP-005 group and placebo group (*F*_(1,18)_ = 0.21, *p* = 0.651). Also during days 20–30, no significant difference was found between the total withdrawal sum scores of the SJP-005 group and the placebo group (*F*_(1,18)_ = 0.65, *p* = 0.429). The most frequently observed withdrawal symptoms were hypersensitivity to touch and vocalization. However, for individual symptoms of both the early and late phase, no significant differences were observed between the SJP-005 and placebo group, except for a significant reduction in the number of erections in the late phase after SJP-005 (*F*_(1,18)_ = 5.23, *p* = 0.034).

**Fig. 4:**
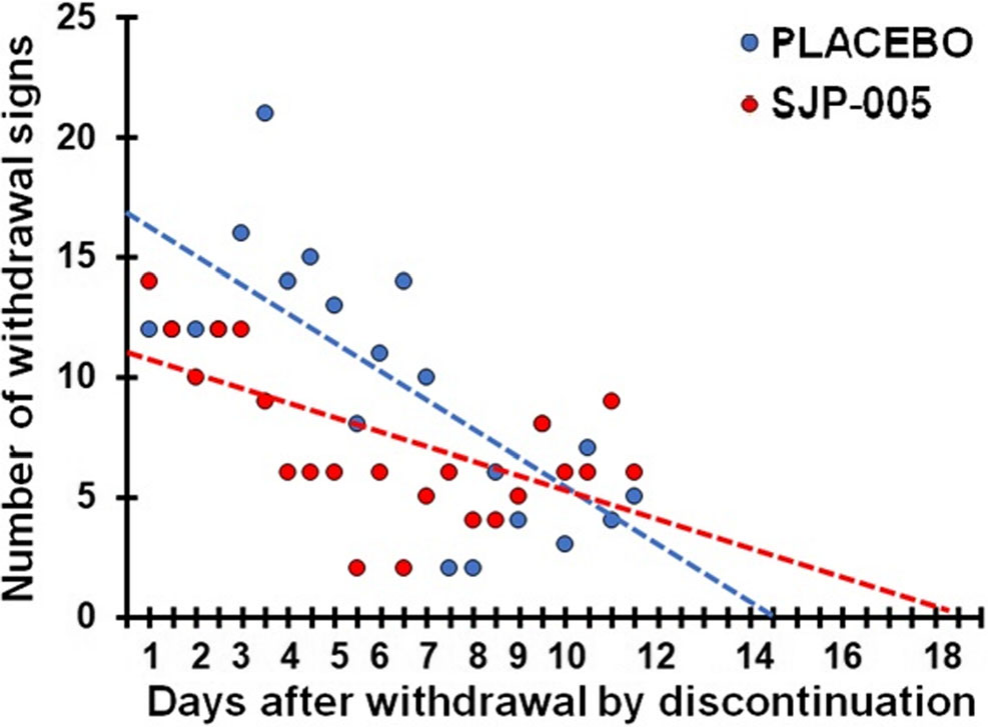
Number of withdrawal signs after morphine withdrawal by discontinuation (T0). Treatment administration started on the same day (day 19) as discontinuation-induced morphine withdrawal (T0). Dots represent the total number of observed withdrawal signs for each group (*N* = 10) for each day; broken lines are lines of best fit for each condition

Regression analysis was conducted on withdrawal scores observed after morphine withdrawal by discontinuation during the late phase (days 20–30). Linear trend lines were computed for the placebo group (*y* = − 1.15 + 16.8) and SJP-005 group (*y* = − 0.57 + 10.7). According to the trendline equations, a 50% reduction in withdrawal symptoms was found 7.3 days after placebo versus 9.4 days after SJP-005. After 14.6 days, all withdrawal symptoms are absent after placebo, while withdrawal symptoms are likely to be expected up to 18.8 days in the SJP-005 group (see Fig. 4). ANOVA revealed that the intercepts (*F*_(1,2)_ = 6.81, *p* = 0.0127) of the trendlines differ significantly between the groups. The slopes do not differ significantly between the placebo and SJP-005 group (*F*_(1,2)_ = 3.68, *p* = 0.0622).

#### Adverse effects and premature death

At the start of the studies, all animals were healthy and displayed no signs of pain, distress or abnormal behavior (by observation). No serious treatment-related adverse effects were observed in the 3 studies. In none of the studies were there significant differences in body weight among animals of the four groups on any test day (see Table 6).

**Table 6:** Mean (SD) body weight (g)

Body weight (g)	Day 1	Day 19	Day 30
Study 1			
Group 1 (placebo)	185 (3.0)	266 (7.7)	299 (9.2)
Group 2 (ketotifen)	186 (4.0)	269 (6.5)	305 (7.1)
Group 3 (ibuprofen)	182 (2.1)	269 (4.6)	314 (5.7)
Group 4 (SJP-005)	189 (2.7)	283 (3.0)	318 (2.1)
Study 2			
Group 1 (placebo)^1^	216 (3.3)	308 (5.3)	343 (8.3)
Group 2 (SJP-005)^1^	218 (1.9)	310 (5.3)	346 (7.0)
Group 3 (placebo)^2^	217 (2.7)	310 (3.9)	343 (4.7)
Group 4 (SJP-005)^2^	214 (2.4)	302 (5.6)	331 (7.1)
Study 3			
Group 1 (placebo)	269 (3.1)	341 (3.0)	363 (8.8)
Group 2 (SJP-005)	263 (2.8)	331 (6.3)	356 (8.3)

No statistically significant changes compared to the placebo group were found

^1^Naltrexone-precipitated withdrawal

^2^Withdrawal by discontinuation

In study 1 and study 3, no differences in food intake were observed among the groups. In study 2, in the naltrexone-precipitated withdrawal groups, food intake on day 24 was significantly higher (*p* < 0.05) after SJP-005. In the discontinuation-induced groups, food intake was significantly higher (*p* < 0.05) after SJP-005 on day 30. These observations are considered random effects, as they were not seen on any other test day.

In study 1, on day 23, one animal was found dead. Gross necropsy revealed an enlarged heart and left lung adhered to rib cage. *N* = 9 animals continued. On day 27, another animal displayed noisy respiration, and an enlarged area on the right thoracic. The animal was not dosed in the afternoon. On day 28 a.m., the animal displayed decreased activity, was cool to touch, head tilt, and had an open wound around the right armpit with brown substance around. The animal’s state was moribund, and as a result prematurely euthanized. The conditions of the two animals were judged as unrelated to the treatment (SJP-005). *N* = 8 animals of group 4 completed the experiment in study 1. In study 2 and study 3, no animals died, nor were any animals sacrificed before completion of the study.

## Discussion

Taken together, the studies revealed that SJP-005 was effective in attenuating the discontinuation-induced morphine withdrawal response in rats when treatment was initiated 2 days before morphine treatment was ceased. The reduction in observed withdrawal symptoms was statistically significantly different from placebo in the late phase (2–12 days after morphine cessation) when SJP-005 treatment was initiated 2 days before morphine cessation.

When administered orally, twice daily starting 4 days before up to 12 days after morphine withdrawal (study 1), SJP-005 treatment was associated with a lower incidence of discontinuation-induced morphine withdrawal response in rats, although the observed effects were not statistically significant in both the early phase (12 h immediately after cessation of morphine) and the late phase (up to 12 days after morphine cessation). Also, SJP-005 appeared to be more effective in discontinuation-induced morphine withdrawal response than the naltrexone-induced morphine withdrawal response. When administered orally immediately after discontinuation-induced morphine withdrawal (study 3), twice daily for up to 12 days, although withdrawal scores were lower after SJP-005, the SJP-005 group did not differ significantly from the placebo group in reducing the incidence of discontinuation-induced morphine withdrawal response in rats.

The studies further showed that animals were withdrawal symptom free at an earlier stage after SJP-005 when compared to placebo (see Figs. 1, 2, and 3). However, this effect was significant when SJP-005 was first administered 2 days before morphine cessation, but reversed when SJP-005 was first administered at the same day of morphine cessation (see Fig. 4). These observations suggest that starting treatment with SJP-005 *before* opioid cessation yields better results compared to initiating treatment after withdrawal started.

The observed effectiveness of SJP-005 in mitigating withdrawal symptoms is in line with current knowledge on the mechanism of action of ibuprofen and ketotifen. Ibuprofen is a well-studied non-steroidal anti-inflammatory agent (NSAID), without abuse potential, and capable of reducing moderate pain with the same clinical efficacy as opioid pain relievers (Apotex, Inc 2020). Ibuprofen reduces pain by inhibiting the cyclooxygenase pathway and reducing prostaglandin production and has also been characterized as a PPAR-γ agonist (Puhl et al. 2015). One study compared ibuprofen to celecoxib (Celebrex) for the reduction of opioid withdrawal pain and opioid craving (Jafari et al. 2017) and found that both ibuprofen and celecoxib showed a marked reduction in pain scores and a reduction in opioid craving. However, ibuprofen was dosed at 400 mg four times a day (1600 mg/day), rather than the higher doses implicated in providing PPAR-γ agonist activity, or maximal anti-inflammatory response (Jafari et al. 2017). Ibuprofen has been prescribed as adjuvant medication for the mitigation of opioid withdrawal symptoms on an “as needed” schedule to reduce pain complaints. However, up to now, it has not been prescribed or investigated for continuous daily use to provide ongoing inhibition of prostaglandin production, and at doses that can provide PPAR-γ agonist activity.

Ketotifen, is a mast cell stabilizing, H_1_-receptor antagonist, and is also a TLR4 inhibitor (Bachtell et al. 2017). This antihistamine drug reduces pro-inflammatory cytokine concentrations, such as interleukin (IL)-1β, IL-6, and tumor necrosis factor-alpha (TNFα), which are increased during morphine administration (Alipour et al. 2018). Ketotifen was tested in mice for its potential as a TLR4 inhibitor to decrease morphine induced tolerance and dependence (Alipour et al. 2018). These animal studies showed that ketotifen reversed morphine tolerance with a single dose, and reduced withdrawal symptoms (weight loss and jumping) with chronic doses. Ketotifen has not been studied in humans for mitigating opioid withdrawal symptoms. However, the antihistaminergic properties of ketotifen have the potential to reduce opioid withdrawal symptoms such as anxiety, nausea and vomiting.

A limiting adverse effect of utilizing NSAIDs for long-term pain relief is the potential damage to the GI mucosa. Ketotifen has been shown to reduce indomethacin (an NSAID)-induced GI mucosa damage in humans (Narendranathan et al. 1999). Therefore, we believe that SJP-005, the combination of ibuprofen and ketotifen, is unlikely to provoke gastrointestinal complaints in significant number of users. Rather, the opposite is expected (i.e., a protective effect).

There are some limitations of the current study that need to be addressed. First, we decided to assess all common withdrawal signs individually, in order to acquire a broad overview of possible withdrawal effects. To enable comparison with other recent studies, we used the same scale as applied in the studies by Hutchinson et al., for example to evaluate ibudilast (Hutchinson et al. 2009). Using this scale, all symptoms contribute equally to the scale sum score. We agree however that it may be important to weight the importance of individual symptoms when calculating a sum score. Unfortunately, the duration and severity of symptoms was not assessed in the current study, and the collected data thus do not allow to transfer scores into weighted scales such as the Gellert-Holtzman scale (Gellert and Holtzman 1978). Future studies could apply weighted scales for this purpose. Second, in the current studies data was only collected whether symptoms were present or not during 2-minute assessment periods. It would have been of interest to also record the frequency and intensity of withdrawal behaviors. As the animal behavior was scored real time and no video recordings were made, it was not possible to re-evaluate the data. It is recommended for future studies to video record the animals and perhaps also expand the 2-min observation period.

In conclusion, the results suggest that SJP-005 is effective in attenuating the discontinuation-induced morphine withdrawal response in rats. The effect was statistically significant in the late phase (2–12 days after morphine cessation) when SJP-005 treatment was initiated 2 days before morphine withdrawal by discontinuation, but did not reach statistical significance when administered 4 days before or at the same time of morphine cessation. Therefore, future research is needed to establish the ideal timing of treatment initiation, and investigate the efficacy of SJP-005 in opioid withdrawal in humans.
